# Herzfrequenzabhängige EKG-Veränderungen bei einem Patienten mit schwerer Sichelzellanämie

**DOI:** 10.1007/s00101-021-00937-4

**Published:** 2021-03-16

**Authors:** Konrad Peukert, Sven Klaschik, Tobias Hilbert

**Affiliations:** Klinik und Poliklinik für Anästhesiologie und Operative Intensivmedizin, Universitätsklinikum Bonn, Venusberg-Campus 1, 53127 Bonn, Deutschland

## Falldarstellung

### Anamnese

Ein 24-jähriger Patient afrikanischer Abstammung mit Sichelzellanämie stellt sich aufgrund eines Low-flow-Priapismus, der auf konservative Behandlung nicht mehr anspricht, zur operativen Notfallanlage eines Al-Ghorab-Shunt vor. Wesentliche Komorbiditäten umfassen eine Noncompaction-Kardiomyopathie mit linksventrikulärer (LV) Dilatation und ein terminales Nierenversagen, welches eine Peritonealdialyse erforderte. Das präoperative Serumkreatinin wird mit 14,5 mg/dl, der Serumharnstoff mit 118 mg/dl sowie das Kalium mit 4,3 mmol/l bestimmt. Ferner findet sich ein Laktatdehydrogenase(LDH)-Wert von 967 U/l, die Lebertransaminasen werden normwertig bestimmt, das Haptoglobin liegt unterhalb der Nachweisgrenze. Es liegt ein ca. 2 Monate alter kardiologischer Befund mit Echokardiographie vor, hier werden eine leicht- bis mittelgradig reduzierte linksventrikuläre sowie eine gute rechtsventrikuläre Pumpfunktion beschrieben, aufgrund der LV-Dilatation zeigt sich eine Mitralklappeninsuffizienz °II. Trotz einer ausgeprägten Anämie mit einem präoperativ gemessenen Hämoglobin(Hb)-Wert von 3,5 g/dl (Hämatokrit 10 % [Befund der venösen Blutgasanalyse (BGA) im OP-Trakt]) stellt sich der Patient klinisch unauffällig dar, insbesondere fehlen klinische Anzeichen einer akuten vasookklusiven Krise. Da zuvor irreguläre antierythrozytäre Antikörper bestimmt worden waren, wird zunächst auf die Transfusion von Erythrozytenkonzentraten (EK) verzichtet. Es erfolgt eine der Situation angemessene Risikoaufklärung. Ein neuroaxiales Anästhesieverfahren lehnt der Patient ab. Nach Installation des Routine-Monitorings (5-Pol-EKG, Pulsoxymetrie, nichtinvasive Blutdruckmessung) wird eine Vollnarkose nach Klinikstandard mit Remifentanil und Propofol eingeleitet, und nach neuromuskulärer Blockade mit Rocuronium wird die endotracheale Intubation durchgeführt. Bei Fehlen klinischer Herzinsuffizienzzeichen sowie einer geplanten Operationsdauer von maximal 1 h wird auf ein invasives Kreislaufmonitoring verzichtet. Insgesamt stellt sich die Anästhesieeinleitung als unauffällig dar. Im Weiteren wird die Narkose als balancierte Anästhesie mit Isofluran und Remifentanil aufrechterhalten.

### Klinischer Befund

**Figure 1:**
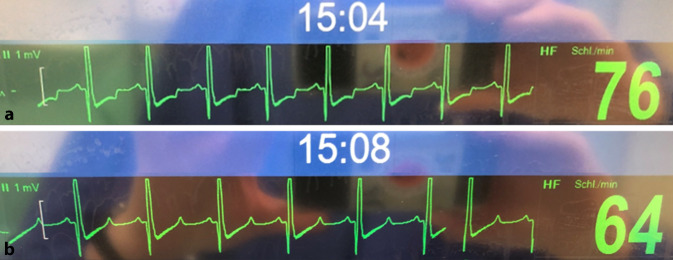

Während des gesamten Eingriffs sind keine Vasopressoren oder Inotropika erforderlich, da der Kreislauf stets stabil ist. Als jedoch die Herzfrequenz mit Beginn der operativen Maßnahmen ansteigt, zeigt das Monitor-EKG des Patienten plötzlich neu auftretende präterminale ST-Segment-Senkungen an, was auf eine relative Myokardischämie schließen lässt (Abb. 1a). Diese Veränderungen scheinen eindeutig herzfrequenzabhängig zu sein, da sich das EKG spontan normalisiert, als die Narkose durch Erhöhung der Opioidapplikation vertieft wird (Abb. 1b). Trotz in der BGA dokumentiertem unverändertem Hb-Wert von 3,5 g/dl sieht sich der behandelnde Anästhesist dennoch nach diesem Ereignis veranlasst, ein kompatibles EK zu transfundieren, was den Hb-Wert auf 5,0 g/dl erhöht.

### Verlauf

Im weiteren Verlauf der Operation treten keine weiteren EKG-Veränderungen mehr auf. Nach der Extubation zeigt der Patient keine klinischen Zeichen einer kardialen Symptomatik. Das Serum-Troponin T ist erhöht (89,6 ng/l [Referenzwert 14 ng/l]), was jedoch aufgrund des Nierenversagens im Endstadium als klinisch nicht relevant erachtet wird. Eine Kontrolluntersuchung zeigt einen stabilen Troponinverlauf; weitere kardiologische Abklärungen werden von dem jungen Patienten explizit abgelehnt. Der Hb-Wert ist bei einer Bestimmung 3 Tage nach dem Eingriff bereits wieder auf einen Wert von 3,7 g/dl abgefallen. In konsiliarischer Zusammenarbeit mit der internistischen Abteilung wird eine Therapie mit Hydroxycarbamid begonnen. Die weiteren oben genannten Laborparameter verändern sich im Verlauf des Klinikaufenthaltes nicht wesentlich, so verbleibt die LDH auf einem moderat erhöhten Niveau, das Haptoglobin weiterhin unterhalb der Nachweisgrenze. Zwei Monate nach der Operation zeigt sich im Rahmen eines Belastungstests echokardiographisch eine anteroseptale Hypokinese, mutmaßlich zurückzuführen auf eine oder mehrere stattgehabte Ischämien bei schwerer Anämie, möglicherweise verstärkt durch die kardiale Grunderkrankung. Eine weitere invasive Koronardiagnostik wird erneut abgelehnt. Unabhängig von der berichteten Episode stellt sich der Patient 4 Monate nach der Operation mit einer Perimyokarditis mit einer Troponin-T-Erhöhung von in der Spitze 1361 ng/l erneut vor.

## Diskussion

Die Sichelzellanämie (SCD) ist eine vererbte monogene Hämoglobinopathie, die durch eine hämolytische Anämie aufgrund der Fehlform der roten Blutkörperchen gekennzeichnet ist. Typisch sind vasookklusive Krisen mit begleitender Gewebe- und Organschädigung. Pro Jahr werden weltweit ca. 300.000 Menschen mit SCD geboren, sodass 2017 in der EU 2,6 von 10.000 Menschen betroffen waren [1]. Bis heute ist die Transplantation hämatopoetischer Stammzellen die einzige kurative Therapieoption. Akute Schmerzen infolge des krisenhaften Verschlusses kleiner Blutgefäße mit nachfolgender Gewebeischämie sind der häufigste Grund für Notfallhospitalisationen [2]. Eine zielgerichtete Therapie solcher Gefäßverschlüsse ist entscheidend, um chronische Dysfunktion und Endorganversagen zu vermeiden.

Priapismus, definiert als verlängerte (>4 h) und schmerzhafte Erektion des Penis, die nicht auf sexuelle Stimulation zurückzuführen ist, betrifft bis zur Hälfte aller männlichen SCD-Patienten und stellt daher eine typische Komplikation dar [3]. Inzwischen als getrennte Entitäten angesehen, beschreiben der ischämische (d. h. venookklusive oder Low-Flow‑) und der nichtischämische (d. h. arterielle oder High-Flow‑)Priapismus zwei verschiedene Formen, jede mit ihrer eigenen Pathogenese. Im Falle der SCD ist der Low-Flow-Priapismus die Hauptmanifestation. Blut wird in den Schwellkörpern (Corpora cavernosa) eingeschlossen, was zu einem extrem schmerzhaften Kompartmentsyndrom führt. Da eine anhaltende Ischämie neben Schmerzen auch zu Nekrosen und Fibrosen der glatten Muskulatur führt, die letztlich eine dauerhafte erektile Dysfunktion zur Folge haben, gilt der Priapismus bei SCD-Patienten als urologischer Notfall. Bei Versagen einer konservativen Therapie (intrakavernöse Injektion von α‑Rezeptor-Agonisten) müssen operative Maßnahmen ergriffen werden.

Kardiale bzw. thorakale Symptome bei SCD-Patienten stellen für den Kliniker oft eine besondere Herausforderung dar. Die Patienten leiden aufgrund von Hämolyse und Milzsequestrierung häufig an einer chronischen Anämie mit Hb-Werten von 8,0 g/dl oder darunter [2]. Wie der hier vorgestellte Fall zeigt, können stark erniedrigte Hb-Werte Anzeichen eines akuten Koronarsyndroms vortäuschen [4]. Aufgrund von Adaptation und der Tatsache, dass das krankhafte HbS eine geringere Sauerstoffaffinität als HbA hat, ist die Gewebeoxygenierung tatsächlich jedoch oft besser als erwartet. Dennoch kann es trotz Fehlens einer tatsächlichen arteriellen Stenose oder gar eines Verschlusses potenziell zu Myokardinfarkten kommen [5]. Bemerkenswert ist dabei, dass die SCD selbst auch ein Risikofaktor für das Entstehen einer Atherosklerose ist [6]. Weitere mögliche Differenzialdiagnosen von Brustschmerzen bei SCD-Patienten umfassen das akute Thoraxsyndrom (das Zusammentreffen von Brustschmerzen, Husten, Fieber, Hypoxie und neu aufgetretenen Lungeninfiltraten) oder eine Lungenembolie.

In dem hier vorliegenden Fall wurden die differenzialdiagnostischen Überlegungen zusätzlich durch das vorbekannte Vorliegen einer Noncompaction-Kardiomyopathie erschwert. Dabei handelt es sich um eine kongenitale Kardiomyopathie, die durch eine echokardiographisch sichtbare Hypertrabekularisierung der Herzmuskulatur durch eine Störung während der embryonalen Organogenese gekennzeichnet ist. Dabei unterbleibt die als Kompaktierung bezeichnete Ausbildung einer regulären Muskelstruktur (daher Noncompaction-Kardiomyopathie). Das klinische Erscheinungsbild variiert, es können Herzinsuffizienzsymptome, thrombembolische Ereignisse im arteriellen Kreislauf sowie Herzrhythmusstörungen vorliegen. Bei dem beschriebenen Fall handelte es sich um eine kompensierte Form, was durch den Echobefund sowie die trotz der ausgeprägten Anämie vergleichsweise gute klinische Belastbarkeit illustriert wird.

Typisch für die SCD sind vasookklusive Krisen, die in erster Linie durch ausgeprägte Schmerzen auffallen und durch die akut kritisch eingeschränkte Organperfusion nicht selten einen intensivstationären Aufenthalt notwendig machen. Diesen Krisen liegen im Wesentlichen die Verstopfung postkapillärer Venolen durch Adhäsion von Erythrozyten, Leukozyten und Thrombozyten an der Endothelwand mit nachfolgender ante- und retrograder Thrombosierung sowie eine durch im Rahmen der Hämolyse freigewordenes Hämoglobin vermittelte Vasokonstriktion zugrunde [7]. Oft sind diese Krisen getriggerte Ereignisse bei einer bei SCD-Patienten vorliegenden chronisch-entzündlichen Vaskulopathie und führen im Verlauf der Erkrankung zu progredienter Organdysfunktion (wie im vorliegenden Fall einer terminalen Niereninsuffizienz oder einer dilatativen Kardiomyopathie). Daher ist eine aggressive Therapie entscheidend. Therapeutische Maßnahmen akuter vasookklusiver Krisen umfassen Transfusionen (vollständiger Austausch- oder einfache („Top-up“-)Transfusion), die Gabe von Kortikosteroiden, Immunglobulinen und Hydroxycarbamid sowie die Behandlung der resultierenden Organdysfunktion [7]. Einen neuartigen Ansatz zur Therapie der schädlichen Wirkung freien Hämoglobins stellen neutralisierende Proteine („*scavenger*“) dar [8].

Dem klinischen Erscheinungsbild sowie den erhobenen Laborbefunden zufolge lag im vorgestellten Fall jedoch keine akute vasookklusive Krise vor.

Während einer akuten SCD-Krise können die systemischen Hb-Werte weiterabnehmen, dies stellt jedoch für sich genommen noch keine absolute Indikation für eine Transfusion dar. Im Gegenteil kann ein zu liberales Transfusionsregime eine beginnende Krise durch die Induktion eines kritischen Hyperviskositätssyndroms noch verschlimmern. Darüber hinaus stellen Transfusionen für SCD-Patienten per se ein Risiko für frühzeitige Alloimmunisierung (wie in dem vorliegenden Fall) und Eisenüberladung dar, was spätere Transfusionen erschweren kann. Gemäß den geltenden Empfehlungen sollte die EK-Transfusion bei akutem Thoraxsyndrom, Schlaganfall, Leber- oder Milzsequestrierung, aplastischer Krise, jeder symptomatischen schweren Anämie (z. B. wie im vorliegenden Fall einer durch EKG-Veränderungen angezeigten relativen myokardiale Ischämie) oder, wenn der Hb-Spiegel akut um mehr als 2,0 g/dl oder unter einen absoluten Grenzwert von ≤ 5,0 g/dl gesunken ist, erfolgen [9]. Die im vorliegenden Fall dokumentierte Grunderkrankung hatte zusätzlichen Einfluss auf die Entscheidung zur Transfusion, da eine Noncompaction-Kardiomyopathie durch die unregelmäßige Muskelstruktur per se zu regionalen Störungen der Myokardversorgung prädisponiert.

Durch eine Transfusion soll beim SCD-Patienten v. a. der prozentuale HbS-Anteil gesenkt sowie die Anämie korrigiert werden. Ziel einer einfachen Transfusion ist es, den Hb-Wert auf das individuelle *Steady-State*-Niveau des Patienten zu normalisieren. Mehrere randomisierte kontrollierte Studien haben sich mit der Frage befasst, ob SCD-Patienten präoperativ transfundiert werden sollten, aber die Ergebnisse sind bislang inkonsistent, weshalb die Entscheidung individuell und unter Beachtung der weiteren Umstände, der spezifischen Kontraindikationen und des Komorbiditätsprofils getroffen werden muss [10, 11].

## Fazit für die Praxis

Stark erniedrigte Hb-Werte bei SCD-Patienten können Symptome einer kardialen Ischämie hervorrufen, und die Diagnosefindung kann eine Herausforderung sein. Dies gilt umso mehr, wenn der Patient in Narkose liegt. Dabei haben SCD-Patienten ein hohes Risiko für vasookklusive Krisen und für ischämiebedingte Gewebe- und Organschäden. Aufgrund krankheitsassoziierter typischer Nebenwirkungen muss die Entscheidung zur Bluttransfusion jedoch auch bei seltener auftretenden sehr stark erniedrigten Hämoglobinwerten immer individuell gefällt werden. Eine gute perioperative interdisziplinäre Zusammenarbeit ist bei der Behandlung dieser Patienten von besonderer Bedeutung.
